# High-Throughput Screening Identifies Idarubicin as a Preferential Inhibitor of Smooth Muscle versus Endothelial Cell Proliferation

**DOI:** 10.1371/journal.pone.0089349

**Published:** 2014-02-24

**Authors:** Shakti A. Goel, Lian-Wang Guo, Bowen Wang, Song Guo, Drew Roenneburg, Gene E. Ananiev, F. Michael Hoffmann, K. Craig Kent

**Affiliations:** 1 Department of Surgery, University of Wisconsin, Madison, Wisconsin, United States of America; 2 Small Molecule Screening & Synthesis Facility, UW Carbone Cancer Center, Madison, Wisconsin, United States of America; Broad Institute of Harvard and MIT, United States of America

## Abstract

Intimal hyperplasia is the cause of the recurrent occlusive vascular disease (restenosis). Drugs currently used to treat restenosis effectively inhibit smooth muscle cell (SMC) proliferation, but also inhibit the growth of the protective luminal endothelial cell (EC) lining, leading to thrombosis. To identify compounds that selectively inhibit SMC versus EC proliferation, we have developed a high-throughput screening (HTS) format using human cells and have employed this to screen a multiple compound collection (NIH Clinical Collection). We developed an automated, accurate proliferation assay in 96-well plates using human aortic SMCs and ECs. Using this HTS format we screened a 447-drug NIH Clinical Library. We identified 11 compounds that inhibited SMC proliferation greater than 50%, among which idarubicin exhibited a unique feature of preferentially inhibiting SMC versus EC proliferation. Concentration-response analysis revealed this differential effect most evident over an ∼10 nM-5 µM window. *In vivo* testing of idarubicin in a rat carotid injury model at 14 days revealed an 80% reduction of intimal hyperplasia and a 45% increase of lumen size with no significant effect on re-endothelialization. Taken together, we have established a HTS assay of human vascular cell proliferation, and identified idarubicin as a selective inhibitor of SMC versus EC proliferation both *in vitro* and *in vivo*. Screening of larger and more diverse compound libraries may lead to the discovery of next-generation therapeutics that can inhibit intima hyperplasia without impairing re-endothelialization.

## Introduction

Atherosclerosis is the leading cause of death in the United States. Interventions for treating atherosclerosis including angioplasty, stenting and bypass frequently fail related to the development of recurrent disease (restenosis) [1]. The pathology of restenosis is primarily intimal hyperplasia, and central to this process is smooth muscle cell (SMC) proliferation. In response to the injury associated with arterial reconstructions, SMCs in the media transform from a differentiated to a proliferative and migratory phenotype leading to the formation of a highly cellular subintimal plaque that re-narrows the vessel lumen [2]. Diminished flow related to narrowed or occluded arteries gives rise to adverse outcomes such as heart attack, stroke, amputation and/or death.

Another key cell type integral to this process is the endothelial cell (EC). As a by-product of interventions to treat atherosclerosis, denudation of the endothelial layer of treated arteries or veins leads to deleterious consequences. First, ECs provide the vessel’s anti-thrombotic lining. Without an EC layer, platelets accumulate on the vessel surface initiating thrombus that can cause sudden death [3]. Equally important, it has been shown that ECs and SMCs interact, such that a uniform EC layer lining the inner surface of a vessel inhibits underlying SMC growth and migration thus lessening the potential for the formation of hyperplastic plaque [4]. Third, an intact EC layer prevents transmigration of leukocytes into the arterial wall and leukocyte infiltration is one of many contributors to the process of intimal hyperplasia [5]. Lastly, recent clinical evidence indicates that endothelial dysfunction produced by rapamycin, a SMC inhibitor used to prevent the development of intimal hyperplasia, leads to impaired collateral flow [5] as well as paradoxical vasoconstriction in the arterial segment adjacent to the rapamycin-releasing stent [6]. Thus, following vascular reconstruction, it is essential that ECs be allowed to rapidly repopulate the vessel lumen [7], [8].

Currently, the only clinically employed method for preventing restenosis is a stent coated with rapamycin or paclitaxel used in conjunction with angioplasty [9]. Unfortunately, both drugs inhibit EC proliferation, migration, and survival and thus impair the critically important process of re-establishing the vessel’s protective endothelial lining [3]. Consequently, despite the success of drug-eluting stents, neo-intima plaque still leads to restenosis in approximately 15% of treated patients [10], [11]. More importantly, impaired re-endothelialization leads to acute or late stent thrombosis which is associated with a 45% mortality [3]. Although dual antiplatelet therapy is used to reduce the incidence of stent thrombosis, the incidence of thrombosis still remains significant (1.3%), and antiplatelet agents are associated hemorrhage and additional cost in this patient population.

Thus, the optimal drug to prevent restenosis would be one that selectively inhibits SMC proliferation and intimal hyperplasia but has a minimally inhibitory effect on EC proliferation. Several such selective agents have been reported in the literature [12], [13] [14]–[16] [17]–[19] but with various limitations, *e.g.* lack of effect *in vivo* or difficulty in delivery. Moreover, the scarceness of reports identifying agents that selectively inhibit SMCs versus ECs likely reflects the fact that ECs are generally more susceptible than SMCs to anti-proliferative drugs. Thus, in order to discover candidates for selective SMC inhibition, a high throughput screening campaign is necessary to screen large libraries of compounds. To the best of our knowledge, there has been a lack of such HTS studies with this goal in mind.

The purpose of this study is to establish a pilot HTS system that is amenable for screening larger chemical libraries with the goal of discovering new drugs that selectively retard human SMC proliferation while leaving the growth of endothelial cells unaffected. To circumvent the shortcomings in the aforementioned studies, we have developed accurate assays for proliferation of primary human aortic SMCs and human aortic ECs, which are more relevant to human restenotic disease than animal cells. In a pilot screen we have identified that idarubicin, an FDA-approved drug currently used for treating leukemia, inhibits the proliferation of SMCs to a much greater extent than ECs through a defined concentration window. Furthermore, for the first time, this drug has been shown through *in vivo* testing to be a potent inhibitor of injury-induced intimal hyperplasia. Our finding raise the possibility of repurposing idarubicin for the treatment of vascular restenosis and establish an efficient, low cost HTS that can be used through screening of large chemical libraries, to identify other candidate inhibitors of restenosis.

## Materials and Methods

### Ethics Statement

The experiments involving animal use were carried out in strict accordance with the recommendations in the Guide for the Care and Use of Laboratory Animals of the National Institutes of Health. The protocol (Permit Number: M02273) was approved by the Institutional Animal Care and Use Committee (IACUC) of the University of Wisconsin-Madison. All surgery was performed under isoflurane anesthesia, and all efforts were made to minimize suffering.

### Materials

Alamar Blue was purchased from Invitrogen (Carlsbad, CA). Cell Titer Glo was from Promega (Madison, WI). Primary human aortic smooth muscle cells (HuAoSMCs) and primary human aortic endothelial cells (HuAoECs) at passage 3 were purchased from Lonza; their respective optimal culture media (SmGM-2 and EGM-2) were from the same commercial source. Cells were used at passage 5 after expansion. Trypsin/EDTA solution was from Clonetics (Walkersville, MD); and DPBS was from Gibco (Invitrogen, Carlsbad, CA). Microtiter tissue 96-well culture plates with transparent flat-bottoms and black-walled sides were from Costar (Corning, NY). Resveratrol and idarubicin were products of Sigma-Aldrich (St. Louis, MO). Stock solutions of these reagents were prepared in DMSO (Thermo-Fisher). The library of NIH Clinical Collection composed of 447 unique compounds of known bioactivity was available at the Small Molecule Screening and Synthesis Facility (SMSSF) of the University of Wisconsin Carbone Cancer Center (UWCCC).

### Cell Culture

Cryo-protected frozen HuAoSMCs and HuAoECs (Lonza, passage 3) were thawed and cultured in their respective media that are optimized for cell growth by the manufacturer. HuAoSMCs were grown in the SmGM-2 medium containing 5% FBS, and HuAoECs in the EGM-2 medium containing 2% FBS in a humidified incubator at 37°C with 5% CO_2_. After expansion, cells at passage 5 were used for all the experiments.

### Test of the Consistency of an Automated Cell Proliferation Assay System

Freshly collected HuAoSMCs (passage 5) were counted (>93% viability) by Cellometer AutoT4 (Nexelon Bioscience), and dispensed using Microflo Select (BioTek) to a final density of 2700 cells/200 µl/well in the SmGM-2 medium in a 96-well plate. After a 24 h incubation to allow cell attachment, 0.1 µl of DMSO (vehicle) or 0.1 µl of resveratrol (a known SMC growth inhibitor) stock in DMSO was robotically transferred using Biomek FX (Beckman) from a resveratrol stock plate into cell culture (final 50 µM resveratrol [20]. DMSO and resveratrol were added into alternate columns of wells (8 wells per column). We used a noncytotoxic and inexpensive reagent (Alama Blue) for proliferation assay. After incubation with resveratrol for 72 h, Alamar Blue dye was added using Matrix Hydra (Thermo-Fisher) and incubated with cells for another 24 h, and fluorescence was then determined using a Safir2 plate reader (Tecan, excitation/fluorescence: 530 nm/590 nm, bandwidth: 15 nm). The data from 40 wells of vehicle and 40 wells of resveratrol treatments were analyzed for assessment of well-well consistency in the assay. Background signal from the cell-free wells (medium only) was subtracted. In agreement with previous studies [21] we found that reading Alamar Blue fluorescence 24 h after incubation reduced variance compared to reading after shorter incubation (*e.g*. 4 h). To verify assay consistency with a additional method, Alamar Blue dye was removed and wells were gently washed, and Cell Titer Glo reagent was then added followed by a 10 min incubation and luminescence measured using Genios Pro.

### HTS against the NIH Clinical Collection using HuAoSMCs and HuAoECs

The HTS assay of cell proliferation was performed to screen 447 compounds included in the NIH Clinical Collection using total six 96-well plates. Each compound was tested once by the addition of 0.1 µl of 10 mM stock dissolved in DMSO to yield a final concentration of 5 µM. Each plate contained 8 wells of negative controls added with DMSO (0.1 µl, final 0.05%) and 8 wells of positive controls added with resveratrol (final 50 µM). HTS assays against the same NIH Clinical Library were conducted with either SMCs or ECs. Cell growth, robotic liquid handling, and fluorescence reading were conducted as described in the preceding paragraph except that the test compounds were transferred (using Biomek FX) from preconfigured stock plates. Robustness of a HTS assay is estimated by the Z′ value [22], which is calculated using the formula: Z^′^ = 1−[3sdc^+^+3sdc^−^)/(mc^+^−mc^−^)] where sd = standard deviation; m = mean; c^+^ = positive control (resveratrol); c^−^ = negative control (DMSO). A Z′ value of 0.5 is considered the minimal robustness for an assay to perform well in HTS [22].

### Dose Responses of Proliferation of HuAoSMCs and HuAoECs

In order to compare differential effects of idarubicin on the proliferation of SMCs versus ECs, dose response experiments were carried out using the two cell types on the same 96-well plate. Assays were performed as described above except that idarubicin (or resveratrol) was added at serial dilutions into triplicate wells for each concentration. Curve fitting was performed with the Prism software (GraphPad).

### Rat Balloon Angioplasty Model and Perivascular Drug Delivery

Balloon injury of the left common carotid artery was performed in Male Sprague-Dawley rats (300–350 g) following our previously described method [23]. Briefly, after induction of anesthesia with isoflurane, a 2F balloon catheter was inserted through the left external carotid artery into the common carotid artery, insufflated at 2 atm of pressure, pulled back to the bifurcation, and repeated 3 times. The external carotid artery was then ligated, and blood flow was resumed through the common and internal carotid arteries.

Immediately after re-establishment of flow, idarubicin (50 µg) or DMSO (vehicle, final 0.1%) dissolved in 300 µl of 25% F127 pluronic gel (Sigma-Aldrich) was applied around the injured segment of the carotid artery (5 animals in each group). The pluronic gel is a biodegradable polymer, which is soluble in water at 4°C but becomes a gel when in contact with tissues at 37°C [24]. Rats were euthanized 14 days after injury, and the injured segments of common carotid arteries were collected and fixed in 4% paraformaldehyde overnight for embedding in paraffin.

### Morphometric Analysis of Intimal Hyperplasia and Restenosis

Nine evenly-spaced sections through each injured carotid artery were stained using routine hematoxylin and eosin (H&E) and images were collected with light microscopy. Intimal and medial areas, and circumferences were determined by measuring the internal elastic lamina (IEL), external elastic lamina (EEL), and lumen for each section using the ImageJ software (National Institutes of Health) [23], [25]. Intimal hyperplasia is quantified by the area ratio of intima versus media; the extent of restenosis is reflected by a reduction in residual lumen [26], a ratio of intimal area versus IEL area.

### Immunostaining of CD31, an Endothelial Cell Marker

To assess re-endothelialization, immunostaining of CD31 (an EC marker) was performed on carotid sections following our previous report. Briefly, a goat anti-CD31 primary antibody (R&D Sytems, 1∶150) was incubated with the sections for 1 h followed by an incubation with a biotinylated rabbit-anti-goat secondary antibody for 30 min. Immunostaining of CD31 was then visualized by using streptavidin-HRP and DAB. Re-endothelialization was quantified following previously published methods [27], [28]. Briefly, the percentage of the luminal perimeter that stained for CD31 versus total perimeter was measured using NIH Image J. Re-endothelialization was then scored from 1 to 5 (1: <20%; 2∶20 to 40%; 3∶40 to 60%; 4∶60 to 80%; 5∶80%–100%) and the scores were averaged with the data from 5 rats (6 sections per rat) in each treatment group.

### Statistical Analysis

All data are presented as mean ± standard error (SEM). Statistical analysis was performed using two-tailed unpaired Student’s t-test. Data are considered statistically significant when a *P* value is <0.05.

## Results

### Development of a HTS System for the Evaluation of Human Vascular Smooth Muscle Cell and Human Vascular Endothelial Cell Proliferation

In order to establish a HTS system that produces consistent outcomes, we first set out to optimize assay conditions. We chose proliferation as an assay since SMC proliferation is the central event in the development of intimal hyperplasia and EC proliferation is essential to re-endothelialization of the vessel lumen and prevention of thrombosis. We chose to use primary human SMCs and ECs so that our findings would be readily translatable; human primary cells are most relevant to human diseases. For the proliferation assay we chose Alamar Blue which has been widely used for measurement of cell number as a surrogate of proliferation. This reagent is easy to use, noncytotoxic, inexpensive [29], and has been previously successfully applied in HTS projects. Moreover, this assay does not require washing or cell lysis thereby minimizing variability. To maximize cell growth rate, we used the complete SmGM-2 medium (supplemented with 5% serum) and EGM-2 medium (2% serum). Both are media optimized by Lonza for HuAoSMCs and HuAoECs, respectively. In preliminary studies, we varied seeding density and assay duration in order to maximize proliferation. We found that SMCs seeded at a density of 2000–3000 cells per well gave rise to maximal proliferation (Figure S1); higher seeding densities did not result in more significant growth (data not shown). Thus, we used for our HTS system a cell density of 2700 cells per well and an assay duration of 72 h. Optimization experiments were also performed for ECs resulting in a similar protocol except for the use of the EGM-2 medium and 2% serum (data not shown). Cell number was determined by quantifying fluorescence from the Alamar Blue reagent.

**Figure 1:**
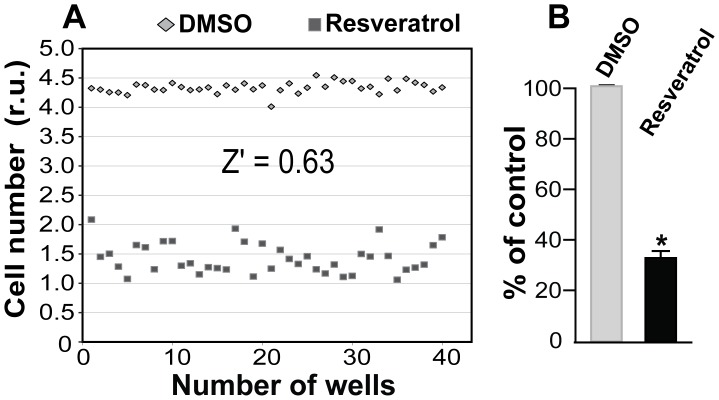
Test of reproducibility of the automated 96-well proliferation assay format. Experiments were performed as described in detail in Materials and Methods. DMSO (40 wells) and resveratrol (40 wells) were used as negative control and positive control, respectively. Data are presented either as Alamar Blue fluorescence reading from individual wells (A), or a mean ± SD (standard deviation) of 40 wells (B) (***P<0.001).

We then applied these optimized conditions to test the consistency of our automated HTS system for proliferation using resveratrol as a positive control. We chose resveratrol because this natural compound has been previously shown to inhibit SMC proliferation and angioplasty-induced intimal hyperplasia [20]. As shown in Figure 1, compared to vehicle control (DMSO), resveratrol inhibited SMC growth by ∼65% with a very small SEM, indicating low well-to-well variation. The good well-to-well consistency achieved by this automated assay format with 96-well plates is also demonstrated by a Z′ value of 0.63. The Z′ factor is generally accepted as a measure to quantify the quality and hence suitability of a particular assay for use in a full-scale, high-throughput screen [22]. A Z′>0.4 is considered a good assay [22]. We also corroborated the Alamar Blue assay results using another established method, Cell Titer Glo assay, which quantifies ATP levels in cell lysates [30]. Cell Titer Glo assays using resveratrol for both SMCs and ECs also produced high Z′ values (>0.7).

### HTS of the NIH Clinical Collection Produces 11 Compounds that Inhibit HuAoSMC Growth Greater than 50%

**Figure 2:**
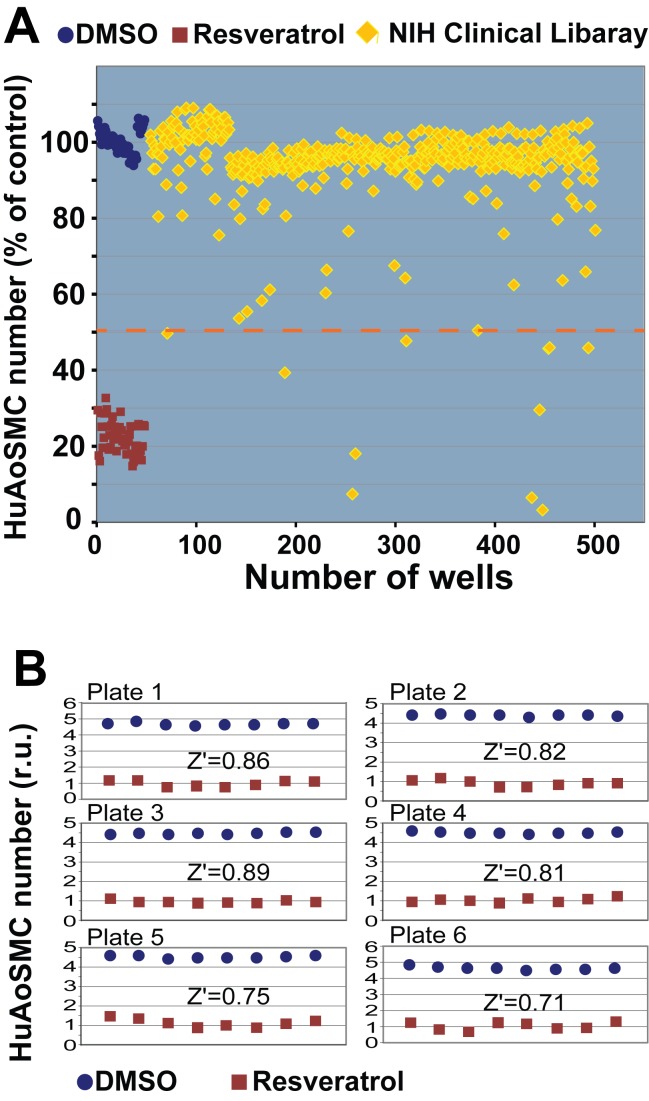
HTS against the NIH Clinical Collection for HuAoSMC proliferation. Assays were performed with SMCs using the automated assay system as described in detail in Materials and Methods. DMSO (blue, final 0.05% in each of 8 wells) and resveratrol (red, final 50 µM in each of 8 wells) served as negative control and positive control, respectively, on each of six 96-well plates. Total 447 compounds in the NIH Clinical Collection (yellow) were tested at a final concentration of 5 µM (1 well for each drug). *For confirmation of the hits with a different method (Cell Titer Glo), please see Figure S2.* **A**). Percent Alamar Blue fluorescence reading. The dashed line marks 50% inhibition of SMC proliferation. **B**). Consistency of HTS assay on each of six 96-well plates.

We then utilized this HTS system to screen 447 FDA-approved drugs in the NIH Clinical Collection. All the compounds included in this collection are clinically used drugs with diverse bioactivities. We used a compound concentration of 5 µM. Six 96-well plates were used with 8 wells of negative control (vehicle, DMSO) and 8 wells of positive control (resveratrol) per plate (Figure 2A). The overall signal to background ratio was 5.1±0.4 for all plates. A Z′ value for each individual plate was calculated using the mean and SD (see Experimental Procedures) from the negative and positive controls. All of the Z′ values were in the range of 0.71–0.89 (Figure 2B). The overall Z′ calculated with the data from all six plates was 0.73. Thus the well-to-well and plate-to-plate consistency was high.

Among the 447 tested drugs, 11 inhibited human SMC proliferation more than 50%, producing a hit rate of ∼2.5% (Figure 2A). We assumed that drugs providing more than 50% SMC inhibition have the greatest likelihood of inhibiting intimal hyperplasia. We then used the orthologous Cell Titer Glo assay to confirm the 11 positive hits. After removal of the Alamar Blue dye, wells were washed gently, and then subjected to the Cell Titer Glo assay. As shown in Figure S2, the Cell Titer Glo assay produced a pattern of inhibition that was similar to Almar Blue.

### Idarubicin Preferentially Inhibits HuAoSMC Versus HuAoEC Proliferation

**Figure 3:**
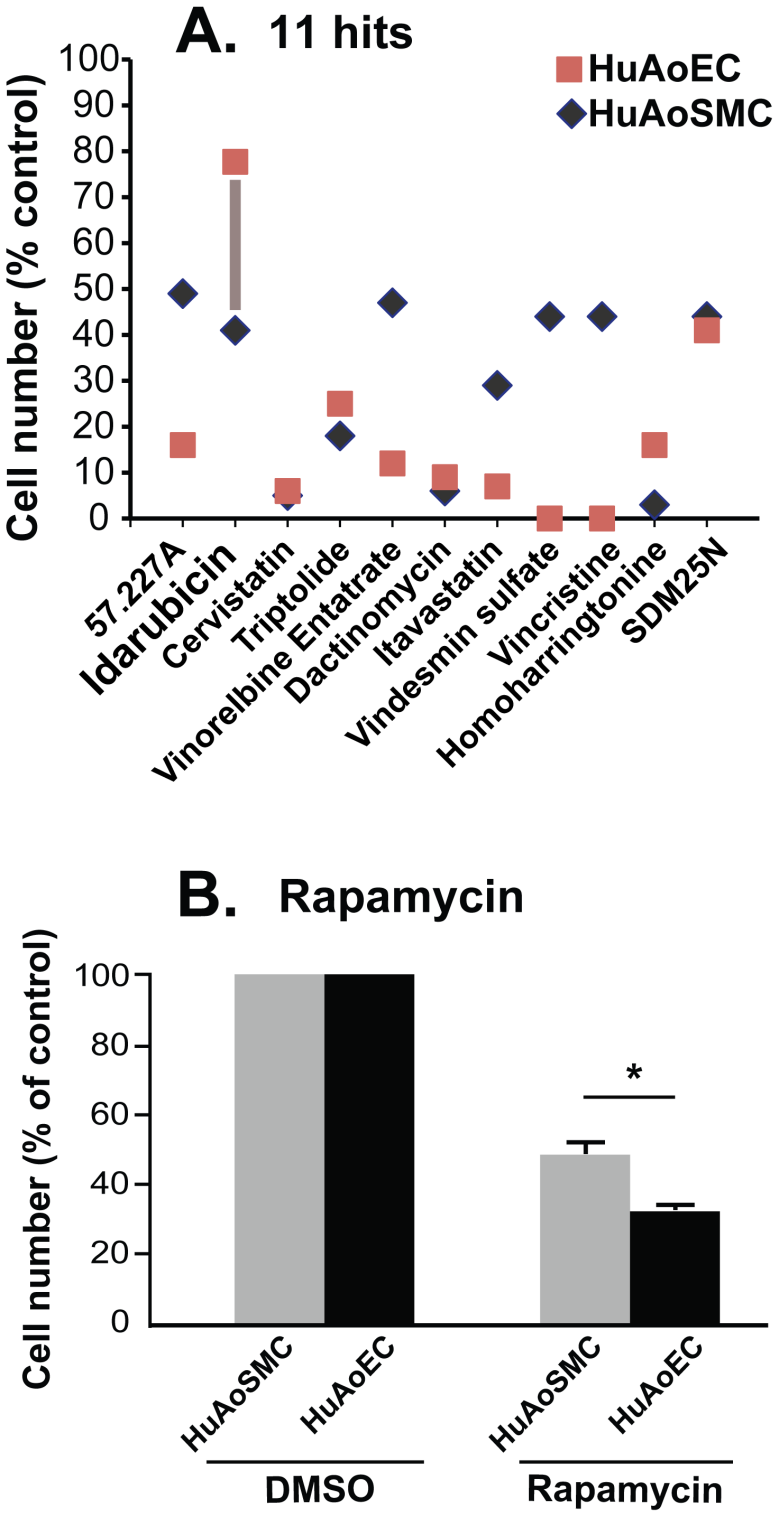
Differential inhibition of HuAoSMC versus HuAoEC proliferation by the 11 hits selected from the NIH Clinical Collection. **A).** HTS of the NIH Clinical Collection was performed using SMCs as well as ECs, as described for Figure 2. Percent inhibition of SMC proliferation (Black) by the 11 hits was compared with that of EC proliferation (red). The vertical bar highlights greater inhibition of SMC versus EC proliferation by idarubicin, which is opposite to the effect of most of the other hits. **B).** Inhibition of cell proliferation by rapamycin was compared between HuAoSMCs and HuAoECs. The experiment was performed using the automated assay system as described in Figure 1. Rapamycin was added to a final concentration of 200 nM. Cell number was assessed by Cell Titer Glo assay. Each bar represents a mean ± SD (*P<0.05).

**Figure 4:**
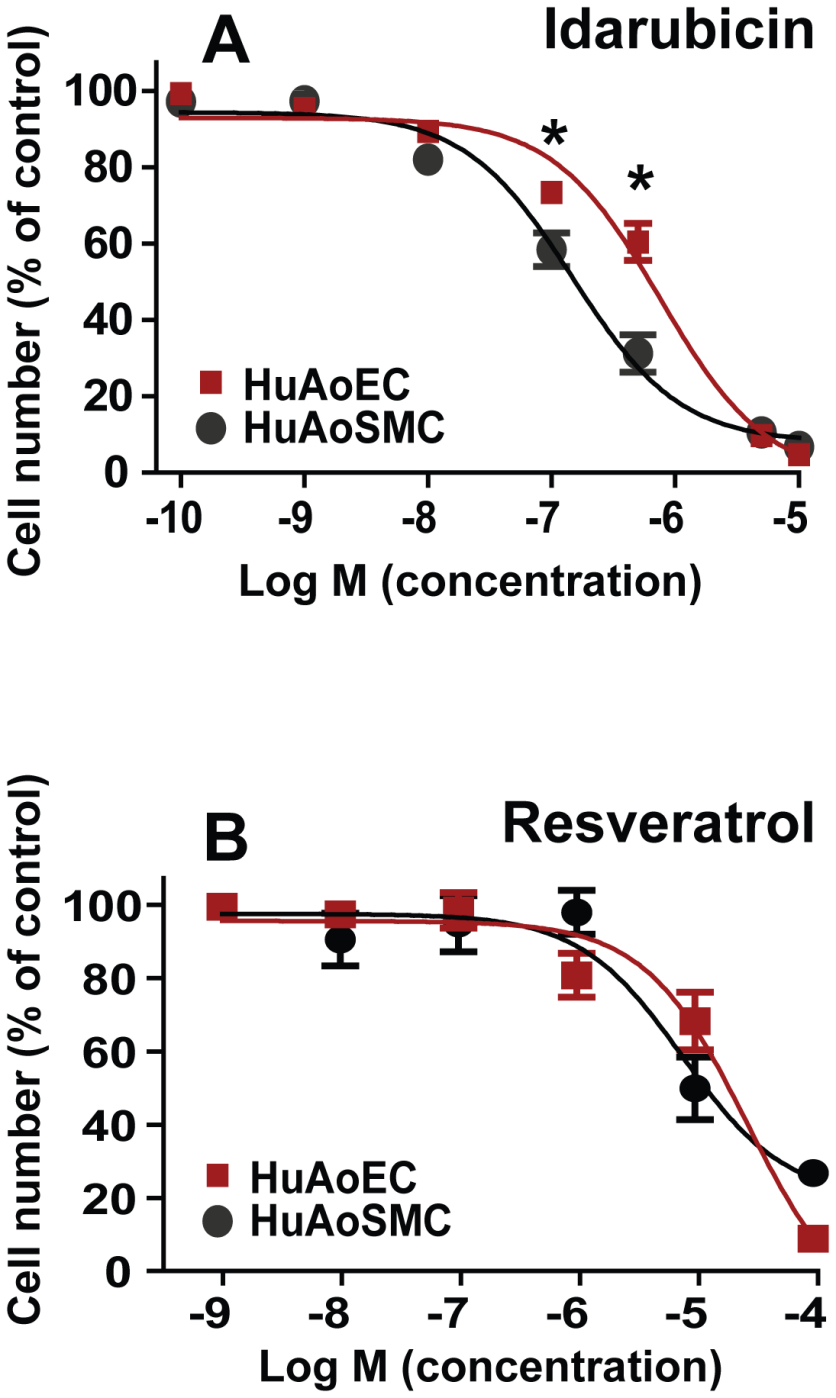
Dose-responses of HuAoSMCs and HuAoECs to idarubicin treatment. Proliferation of SMCs or ECs in the presence of various concentrations of idarubicin or resveratrol was assayed in a 96-well plate and handled by the same robotic system as described in Materials and Methods. Each data point is a mean ± SD of triplicates, *P<0.05.

To identify drugs that selectively inhibit the growth of SMCs versus ECs, we performed the same HTS assay against the NIH Clinical Collection using ECs. Z′ values for the endothelial assay calculated from six 96-well plates were all >0.7, indicating an excellent consistency. We then compared percent inhibition of EC proliferation to that of SMC proliferation for the 11 hits from the SMC assay. As shown in Figure 3A, 5 of the compounds inhibited ECs more than SMCs. We concurrently also evaluated rapamycin, a cliniclally used inhibitor of intimal hyperplasia (Figure 3B). Consistent with the propensity for rapamycin-coated stents to induce thrombosis secondary to inhibition of re-endothelialization, rapamycin also inhibited EC proliferation to a much greater degree than SMC proliferation. Four of the 11 compounds (cervistatin, triptolide, dactinomycin, and SDM25N) inhibited EC and SMC proliferation to an approximately equal degree. However, two of the 11 compounds inhibited EC proliferation to a lesser degree than that of SMCs. The first of these compounds, homoharringtonine, was associated with a small, approximately 10% advantage for ECs. In contrast to the other 10 hits, idarubucin stood out as a unique drug that demonstrated significant selectivity between SMCs and ECs. That is, idarubicin reduced SMC proliferation by ∼60% but suppressed EC growth by only ∼20% (Figure 3A). Since the HTS assays were conducted at a single drug concentration (5 µM) which is conventionally used for primary screens, we created idarubicin dose-response curves for proliferation of both SMCs and ECs using the protocol from our HTS assays. Resveratrol dose-response curves were also generated for comparison. Blending together dose response curves for the two cell types (Figure 4A) revealed a concentration window of ∼10 nM-5 µM, where idarubicin preferentially inhibited SMC (IC50 = 0.13 µM) versus EC proliferation (IC50 = 0.61 µM). In contrast, within a concentration range of 1 nM–100 µM resveratrol did not produce differential inhibition of SMC versus EC proliferation (Figure 4B).

**Figure 5:**
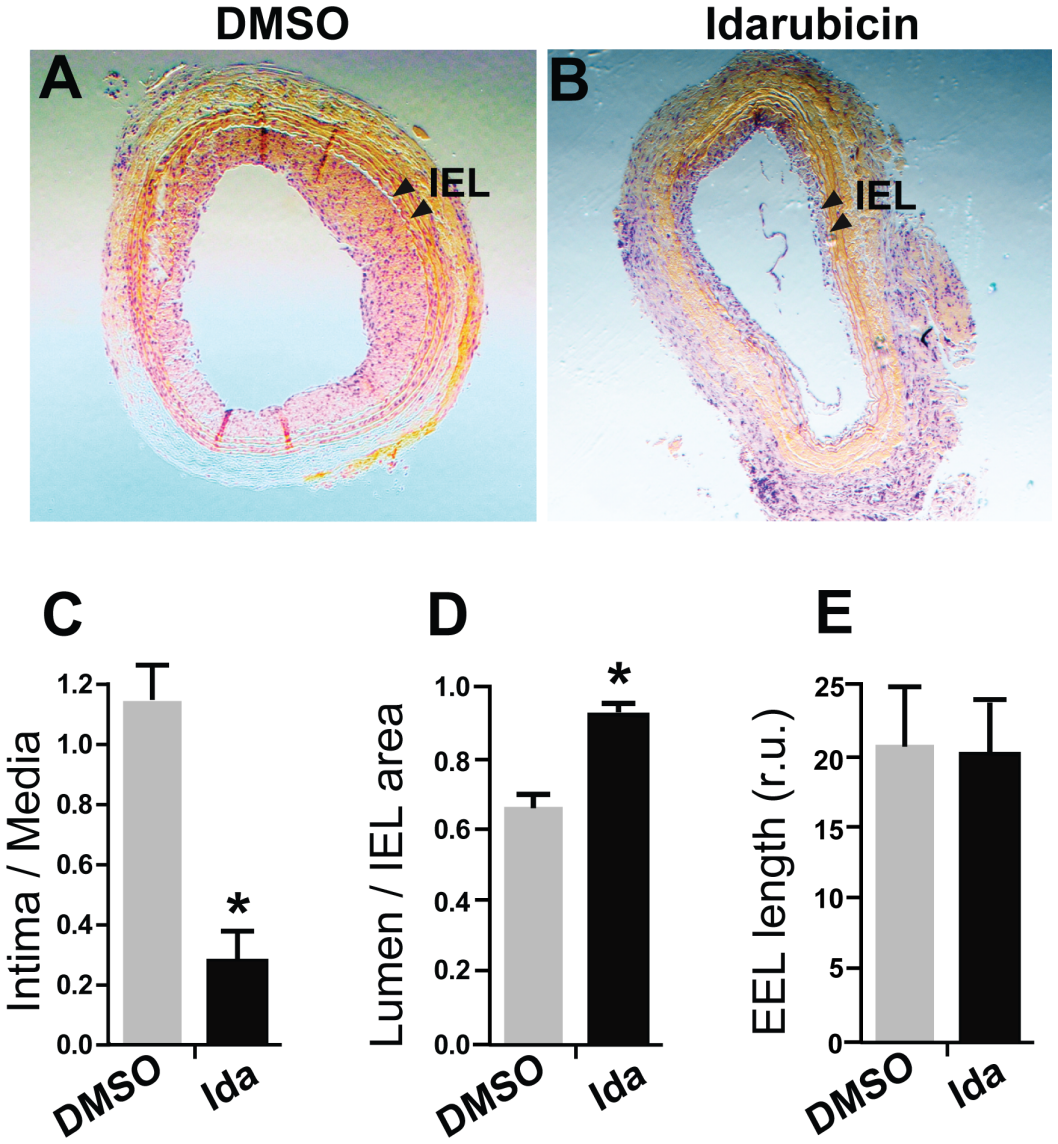
Inhibitory effect of idarubicin on intimal hyperplasia in balloon-injured rat carotid arteries. Following balloon angioplasty, idarubicin was applied locally around the injured arteries. Morphometric analysis was performed on the sections of carotid arteries collected on day 14 post angioplasty, as described in detail in Materials and Methods. Shown in A and B are representative H&E-stained sections from the arteries treated with vehicle (DMSO) and idarubicin, respectively. Arrow heads point to IEL. Statistics of the area ratio of intima versus media (C), residual lumen (the ratio of lumen area versus IEL area) (D), and EEL length (E) were calculated with the data pooled from 5 rats in each treatment group. Each bar represents a mean ± SEM (*P<0.05).

### Locally Administered Idarubicin Inhibits Intimal Hyperplasia but not Re-endothelialization in Rat Carotid Arteries Following Balloon Injury

Idarubincin is a drug used for treating leukemia, but whether it has an inhibitory effect on intimal hyperplasia has not been reported. Prompted by its favorable property of selectively inhibiting SMC proliferation, we evaluated the ability of idarubicin to suppress intimal hyperplasia using an established rat carotid angioplasty model of restenosis (which mimics the post-angioplasty pathology in humans). In order to minimize undesirable side effects that could result from systemic drug delivery, we administered idarubicin locally around the common carotid artery following injury by balloon angioplasty. The morphometric data show that on day 14 after angioplasty, an aggressive neointimal plaque develops (see vehicle control, Figure 5A). However, arteries treated with idarubicin were found to have an 80% reduction in intimal hyperplasia (Figure 5B and C) compared to vehicle control. Moreover, the relative lumen size (calculated as a ratio of luminal area versus IEL area [26]) of arteries treated with idarubicin was substantially increased compared to vehicle control (approximately 45%, Figure 5D). No significant effect of idarubicin on arterial remodeling (EEL length) was observed (Figure 5E).

**Figure 6:**
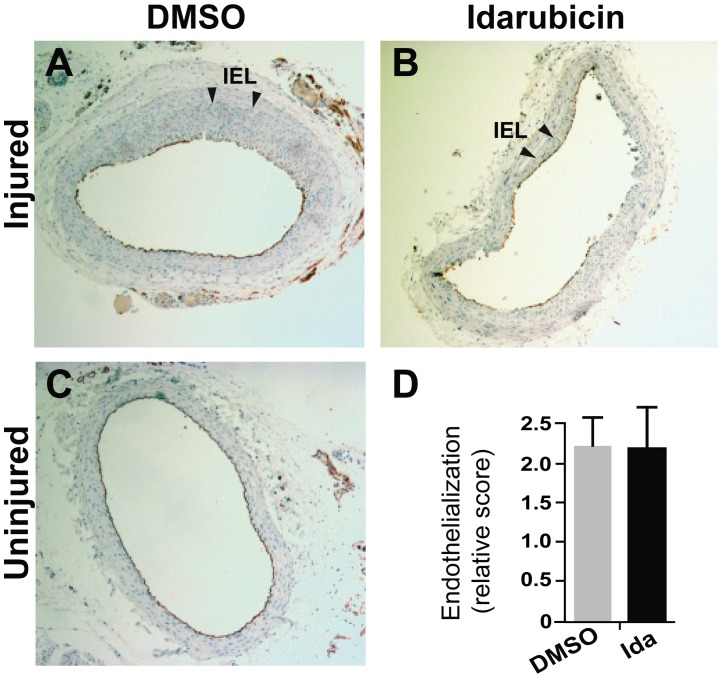
Lack of effect of idarubicin on re-endothelialization in balloon-injured rat carotid arteries. Following balloon angioplasty, idarubicin was applied locally around the injured arteries. For determination of re-endothelialization, immunostaining of CD31 was performed on the sections of carotid arteries collected on day 14 post angioplasty, as described in detail in Materials and Methods. Shown in A and B are representative immunostained sections from the arteries treated with vehicle (DMSO) and idarubicin, respectively. Arrow heads point to IEL. A section of uninjured right carotid artery (C) shows CD31 staining of the undisrupted endothelial layer (see the brown circle). The relative score of re-endothelialization (stained versus total circumference) was quantified with the data pooled from 5 rats in each treatment group (D). Each bar represents a mean ± SEM.

In our *in vitro* experiments idarubicin differentially inhibited SMC proliferation with a lesser effect on ECs (Figures 3 and 4). With this in mind, we further explored whether idarubicin could spare the endothelial layer while attenuating the growth of the neointima. Using the carotid artery sections collected on day 14 following angioplasty, we performed immunostaining for CD31 (Figure 6, A–C), a commonly used marker for assessment of the endothelium. Quantification of CD31 staining indicated that a similar extent of re-endothelialization was achieved in idarubicin-treated arteries compared to that in vehicle-treated arteries (Figure 6D). This result suggests that idarubicin as a potent inhibitor of SMC proliferation and intimal hyperplasia does not impose a significant inhibitory effect on the endothelial recovery after angioplasty denudation.

## Discussion

The anti-restenosis drugs currently available for clinical use inhibit vascular SMC proliferation, migration and survival, but also suppress growth, mobility and survival of ECs [31]. The latter results in adverse side effects, such as delayed re-endothelialization and late stent thrombosis, compromising the long-term efficacy of these treatments [8], [32]. Despite the efficacy of these drugs in preventing restenosis, 15% of patients still develop recurrent disease. The lack of an endothelial lining has been shown to propagate intimal hyperplasia. Thus drugs that do not inhibit re-endothelialization may be more effective in preventing restenosis. The ideal drug designed to prevent restenosis should have a selective anti-proliferative effect on SMCs, but be inert toward ECs. Such candidate drugs could be identified through cellular assay-based high throughput screening (HTS). However, in PubChem or the literature there is a lack of reports of HTS campaigns with the goal of identifying compounds that differentially inhibit SMC versus EC proliferation.

In this study, using relevant primary human SMCs and ECs, we have demonstrated good reproducibility of an automated HTS assay system. We have used this system to screen the NIH Clinical Collection of 447 compounds and identified 11 that produced greater than 50% inhibition of SMC proliferation. Among these hits idarubicin exhibited the unique feature of preferentially inhibiting SMC versus EC proliferation. Moreover, idarubicin had a profound effect on intimal hyperplasia without affecting re-endothelialization, a novel role for a drug that is currently clinically used to treat leukemia. Our study demonstrates the feasibility of using HTS to identify compounds that inhibit the proliferation of SMCs while minimally affecting EC growth. In addition, our HTS format is scalable to large compound libraries, opening the pathway for discovery of additional compounds that differentially affect SMC and EC function.

Vascular SMCs and ECs share many similarities. However, there is also evidence in the literature supporting the existence of pathways and targets that are differentially important to proliferation of SMCs versus ECs. For example, the oligo inhibitors of miRNA-221/222 have been reported to inhibit rat aortic SMC proliferation but stimulate human umbilical vein endothelial cell (HUVEC) growth [18]. Following delivery through intraluminal infusion and also periadventitial administration, these inhibitors were found to suppress post-angioplasty intimal hyperplasia but not re-endothelialization in rats [18]. Different expression levels of the target genes of miR-221/222 in SMCs and ECs might account for differential cellular effects of miR221/222 in these two cell types. In another report, local gene transfer of p85αPKA reduced neointimal formation without affecting endothelial regeneration after balloon injury in rats [33]. The authors found that cAMP inhibits vascular SMC proliferation through the phosphorylation of (Ser83) p85α, which forms an inhibitory complex with p21^ras^, preventing ERK1/2 activation. However, cAMP-induced cell cycle inhibition of ECs is independent of cAMP/PKA modification of p85α. In addition, activation of AMPK has been shown to inhibit SMC proliferation and intimal hyperplasia in a mouse wire injury model while preserving the endothelial layer [34]. Interestingly, expressing AMPK in ECs in a cell type-specific manner stimulates EC growth through up-regulation of HO-1 [35], suggesting differential pathways targeted by AMPK in these two cell types. Aside from differential signaling pathways in SMCs and ECs, differential drug uptake by these two cell types could also explain the varying effects of a particular drug on growth. Since idarubicin inhibits cell growth by intercalating DNA (a universal pathway in all cells) it is likely that differential uptake of idarubicin rather than differential targets/pathways in SMCs and ECs is responsible for the selective inhibition of SMC versus EC proliferation.

These findings suggest that it is possible to identify drugs that differentially inhibit SMCs versus ECs. To this end, we have established a 96-well HTS format through test assays using resveratrol as a control drug and pilot screens using human SMCs and ECs. We began by optimizing cell number, treatment times and liquid handling protocols to maximize the signal to background ratio and Z′ (See Figure S1). The Alamar Blue method has been successfully applied in HTS studies with the major advantage of reducing cost per well [21], [29]. Our findings with Alamar Blue were confirmed with Cell Titer Glo assay, uniformly demonstrating more significant inhibition of SMC proliferation with each of the compounds tested (Figure S2) [30]. Thus for future scale-up screens it is advisable to use Alamar Blue for the primary assay and Cell Titer Glo as the orthologous assay to confirm hits. Although the results reported herewith are with a 96-well format we have recently shown that the assay can be readily converted to a format using 384-well plates [36], [37]. Alternatively, as indicated by the high reproducibility of assays in this study, HTS of larger libraries can be implemented using the 96-well format.

While a HTS format of one well per compound is widely used in the literature, the rationale for using this format in our HTS study is several fold. First, prior to HTS we used 40 wells for each of the positive and negative controls to specifically determine well-to-well variation. A value of Z′ (0.65) above 0.5 indicated an excellent well-to-well consistency. Second, upon HTS assays we again confirmed a low variation between wells on each plate using 8 wells for each of the positive and negative controls (Z′>0.7, Figure 2B). Thus the high Z′s suggest that possible errors in negative or positive hits were minor. Moreover, we performed parallel HTS of the same library with ECs to effectively narrow down the number of hits thus minimizing potential positive hit errors (Figure 3A). Finally, dose response determination provided a definitive measure to confirm a preferential effect of the lead hit (idarubicin) on inhibition of SMC versus EC proliferation in a range of concentrations (Figure 4A).

In our screening of the NIH clinical collection our initial hit rate was 2.5%. Factors contributing to the initial hit rate include final concentration of tested compounds, the chosen library, sensitivity of assay method, and the threshold for selecting hits *etc*. In the primary screen we used a relatively low drug concentration (5 µM) to minimize nonspecific drug effects. In addition, 50% inhibition of SMC proliferation measured by Alamar Blue (equal to ∼80% if measured by Cell Titer Glo, see Figure S2) is a quite stringent threshold. Although we identified only 11 compounds with significant inhibition of SMC proliferation, this rate could easily have been increased by increasing the drug concentration or lowering the threshold or stringency. Another important component of this evaluation is the determination of dose response curves. A given compound’s ability to differentially inhibit SMC and EC proliferation will most likely be dependent upon drug concentration. For most compounds extremely high concentrations are likely to produce cytotoxicity regardless of the cell type. Likewise extremely low concentrations will have only a minimal effect. Thus it is important to search the middle range of concentrations of a given compound for a differential effect on SMC versus EC proliferation. We chose to further evaluate idarubicin because at a concentration of 5 µM there was a differential effect on SMC versus EC proliferation and we found this differential effect persisted through a range of ∼10 nM−5 µM. Other two tested compounds, homoharrinytonine and triptolide, also exhibited a greater inhibition of SMC versus EC proliferation, although the differential effect was only 10% and 3%, respectively. Nevertheless, this differential inhibition may have been more significant at a lesser or greater concentration of these compounds. Thus by creating concentration response curves for all hit compounds one would avoid eliminating hits that inhibit the growth of both cell types at a single concentration but have a differential effect at a concentration other than the one used in the initial screening [38]. Although not necessarily practical for screening large numbers of compounds, the ideal method of evaluating a compound is to perform and compare full concentration response curves for both EC and SMC proliferation.

Through HTS against the NIH Clinical Collection, idarubicin has emerged as an examplary compound demonstrating selective inhibition of SMC versus EC proliferation. Considering that generally inhibitors of proliferation including the clinically used drug, rapamycin, impose a more profound effect on ECs than SMCs (Figure 3B), the findings of a more potent inhibitory effect of idarubicin on SMCs versus EC proliferation is highly desirable. Importantly, in our *in vivo* study idarubicin proved to be effective in reducing intimal hyperplasia in an established rat carotid angioplasty model of restenosis which mimics post-angioplasty pathology in humans. Idarubicin is an analog of daunorubicin with improved properties over other anthracyclines, including higher lipophilicity and hence better cellular uptake. This drug is FDA-approved for treating childhood acute lymphoblastic leukemia. Recently, idarubincin has entered clinical trials for adult patients with acute myeloid leukemia. Although there is evidence of cardiotoxic effects of idarubicin following systemic delivery [39], its use in the prevention of restenosis would be achieved through local delivery (drug-coated stent [10], [40] or balloon [41], [42]). Importantly, despite its profound inhibitory effect on intimal hyperplasia, idarubicin did not have a significant effect on re-endothelialization, which is consistent with our *in vitro* findings demonstrating preferential inhibition of SMC versus EC proliferation (Figure 4A). Thus, further characterization of idarubicin for its potential in treating restenosis is warranted.

While the HTS approach with two human cell types is promising for discovering novel functions of known drugs or potential novel drugs, there are limitations in the current study. A major one is the complexity to translate *in vitro* results into desired *in vivo* outcomes. For example, it is not readily practical to recapitulate the SMC/EC interactions *in vitro* in order to precisely understand their *in vivo* functions or differential responses to drug treatment. Moreover, considering drug diffusion to the greater perivascular space, tissue barriers for drug permeability into SMCs, and drug decomposition over time *etc*., majority of the perivascularly administered drug would not be able to reach SMCs in the vessel wall. Thus an *in vivo* dose in great excess over an effective dose derived from *in vitro* studies may be necessary. Ideally, different amounts of drug would be tested *in vivo* for finding an optimal dose. Even though we have obtained a favorable effect of idarubicin on inhibition of intimal hyperplasia in the rat carotid injury model, it remains a question whether this outcome can be translated to human patients. In future studies, it will be necessary to use a porcine coronary model [42] which is close to human restenotic conditions to further examine the anti-restenotic efficacy of idarubicin. Nevertheless, combined use of our HTS system and an established rat restenosis model constitutes a viable platform for identifying lead compounds that may potentially develop into effective therapeutics.

In sum, using human vascular cells we have established the first HTS format that is adaptable to large-scale screening with a specific goal of discovering novel compounds that selectively inhibit SMC versus EC proliferation. We have demonstrated the validity of this HTS assay, through a screen against the NIH Clinical Library and idarubicin was identified as a selective drug that preferentially suppresses SMC versus EC growth both *in vitro* and *in vivo.* The HTS protocol developed herewith can be used to screen large libraries for compounds that inhibit SMC proliferation with no or reduced effect on ECs. The hits from these screens may generate new compounds that can be translated into therapeutics for the prevention of intimal hyperplasia while allowing re-endothelialization (the desired properties for the next-generation anti-restenotic drugs). Since mechanisms for selective inhibition of SMC versus EC proliferation are not well understood [18], new selective drugs will provide valuable tools for elucidating the intracellular pathways and targets that are differentially important for proliferation of human vascular SMCs versus ECs. Moreover, by screening more diverse libraries we may identify compounds that have properties more favorable than idarubicin, *e.g.* a wider concentration window for selective inhibition of SMCs versus ECs. Ultimately, further screening studies based on our HTS format using human SMCs and ECs will allow the discovery of highly selective and potent small molecule drugs for the purpose of developing safe, efficacious treatments for vascular restenosis.

## Supporting Information

Figure S1 **Time courses of the growth of HuAoSMCs seeded at different densities.** SMCs were seeded at 1000 (blue), 2000 (green), or 3000 (red) cells/well on a 96-well plate, and cultured in SmGM-2 supplemented with 5% serum. Alamar Blue dye was added at different time points (to separate wells) and after a 24 h continued incubation fluorescence was read. A background reading from cell-free wells was subtracted.
(TIF)

Figure S2 **Re-test of the initial hits from the HTS using Cell Titer Glo assay.** Following the HTS assay of HuAoSMC proliferation, Alamar Blue dye was removed and the wells were gently washed by the automated system. The plates were then subjected to Cell Titer Glo assay, and percent inhibition of SMC proliferation by some of the initial 11 hits was compared between these two different assay methods.
(TIF)

## References

[1] Mills B, Robb T, Larson DF (2012) Intimal hyperplasia: slow but deadly. Perfusion 27: 520–528. 22751382 10.1177/0267659112452316

[2] Suwanabol PA, Kent KC, Liu B (2011) TGF-beta and restenosis revisited: a Smad link. J Surg Res 167: 287–297. 21324395 10.1016/j.jss.2010.12.020 PMC3077463

[3] Iakovou I, Schmidt T, Bonizzoni E, Ge L, Sangiorgi GM, et al (2005) Incidence, predictors, and outcome of thrombosis after successful implantation of drug-eluting stents. JAMA: the journal of the American Medical Association 293: 2126–2130. 15870416 10.1001/jama.293.17.2126

[4] Curcio A, Torella D, Coppola C, Mongiardo A, Cireddu M, et al (2002) Coated stents: a novel approach to prevent in-stent restenosis. Italian heart journal: official journal of the Italian Federation of Cardiology 3 Suppl 416S–19S. 12116820

[5] Simon DI (2012) Inflammation and vascular injury. Circulation journal: official journal of the Japanese Circulation Society 76: 1811–1818. 22785436 10.1253/circj.cj-12-0801 PMC4090145

[6] Togni M, Windecker S, Cocchia R, Wenaweser P, Cook S, et al (2005) Sirolimus-eluting stents associated with paradoxic coronary vasoconstriction. J Am Coll Cardiol 46: 231–236. 16022947 10.1016/j.jacc.2005.01.062

[7] Mills B, Robb T, Larson DF (2012) Intimal Hyperplasia: slow but deadly. Perfusion. 10.1177/0267659112452316 22751382

[8] Curcio A, Torella D, Indolfi C (2011) Mechanisms of smooth muscle cell proliferation and endothelial regeneration after vascular injury and stenting: approach to therapy. Circulation journal: official journal of the Japanese Circulation Society 75: 1287–1296. 21532177 10.1253/circj.cj-11-0366

[9] Windecker S, Remondino A, Eberli FR, Juni P, Raber L, et al (2005) Sirolimus-eluting and paclitaxel-eluting stents for coronary revascularization. The New England journal of medicine 353: 653–662. 16105989 10.1056/NEJMoa051175

[10] Mehilli J, Byrne RA, Tiroch K, Pinieck S, Schulz S, et al (2010) Randomized trial of paclitaxel- versus sirolimus-eluting stents for treatment of coronary restenosis in sirolimus-eluting stents: the ISAR-DESIRE 2 (Intracoronary Stenting and Angiographic Results: Drug Eluting Stents for In-Stent Restenosis 2) study. J Am Coll Cardiol 55: 2710–2716. 20226618 10.1016/j.jacc.2010.02.009

[11] Inoue T, Croce K, Morooka T, Sakuma M, Node K, et al (2011) Vascular inflammation and repair: implications for re-endothelialization, restenosis, and stent thrombosis. JACC Cardiovascular interventions 4: 1057–1066. 22017929 10.1016/j.jcin.2011.05.025 PMC3341937

[12] Giordano A, Romano S, Monaco M, Sorrentino A, Corcione N, et al (2012) Differential effect of atorvastatin and tacrolimus on proliferation of vascular smooth muscle and endothelial cells. Am J Physiol Heart Circ Physiol 302: H135–142. 22058159 10.1152/ajpheart.00490.2011

[13] Sun L, Zhao R, Zhang L, Zhang T, Xin W, et al (2012) Salvianolic acid A inhibits PDGF-BB induced vascular smooth muscle cell migration and proliferation while does not constrain endothelial cell proliferation and nitric oxide biosynthesis. Molecules 17: 3333–3347. 22418933 10.3390/molecules17033333 PMC6268737

[14] Vallieres K, Petitclerc E, Laroche G (2009) On the ability of imatinib mesylate to inhibit smooth muscle cell proliferation without delaying endothelialization: an in vitro study. Vascular pharmacology 51: 50–56. 19258052 10.1016/j.vph.2009.02.003

[15] Hacker TA, Griffin MO, Guttormsen B, Stoker S, Wolff MR (2007) Platelet-derived growth factor receptor antagonist STI571 (imatinib mesylate) inhibits human vascular smooth muscle proliferation and migration in vitro but not in vivo. J Invasive Cardiol 19: 269–274. 17541129

[16] Yoon JW, Cho BJ, Park HS, Kang SM, Choi SH, et al.. (2012) Differential effects of trimetazidine on vascular smooth muscle cell and endothelial cell in response to carotid artery balloon injury in diabetic rats. International journal of cardiology. 10.1016/j.ijcard.2011.12.061 22240760

[17] Forte A, Grossi M, Turczynska KM, Svedberg K, Rinaldi B, et al (2013) Local inhibition of ornithine decarboxylase reduces vascular stenosis in a murine model of carotid injury. International journal of cardiology 168: 3370–3380. 23680596 10.1016/j.ijcard.2013.04.153

[18] Liu X, Cheng Y, Yang J, Xu L, Zhang C (2012) Cell-specific effects of miR-221/222 in vessels: molecular mechanism and therapeutic application. J Mol Cell Cardiol 52: 245–255. 22138289 10.1016/j.yjmcc.2011.11.008 PMC3664545

[19] Yao EH, Fukuda N, Ueno T, Matsuda H, Nagase H, et al (2009) A pyrrole-imidazole polyamide targeting transforming growth factor-beta1 inhibits restenosis and preserves endothelialization in the injured artery. Cardiovasc Res 81: 797–804. 19098300 10.1093/cvr/cvn355

[20] Breen DM, Dolinsky VW, Zhang H, Ghanim H, Guo J, et al (2012) Resveratrol inhibits neointimal formation after arterial injury through an endothelial nitric oxide synthase-dependent mechanism. Atherosclerosis 222: 375–381. 22552115 10.1016/j.atherosclerosis.2012.03.021

[21] Nociari MM, Shalev A, Benias P, Russo C (1998) A novel one-step, highly sensitive fluorometric assay to evaluate cell-mediated cytotoxicity. Journal of immunological methods 213: 157–167. 9692848 10.1016/s0022-1759(98)00028-3

[22] Zhang JH, Chung TD, Oldenburg KR (1999) A Simple Statistical Parameter for Use in Evaluation and Validation of High Throughput Screening Assays. Journal of biomolecular screening 4: 67–73. 10838414 10.1177/108705719900400206

[23] Kundi R, Hollenbeck ST, Yamanouchi D, Herman BC, Edlin R, et al (2009) Arterial gene transfer of the TGF-beta signalling protein Smad3 induces adaptive remodelling following angioplasty: a role for CTGF. Cardiovasc Res 84: 326–335. 19570811 10.1093/cvr/cvp220 PMC2761202

[24] Ji R, Cheng Y, Yue J, Yang J, Liu X, et al (2007) MicroRNA expression signature and antisense-mediated depletion reveal an essential role of MicroRNA in vascular neointimal lesion formation. Circ Res 100: 1579–1588. 17478730 10.1161/CIRCRESAHA.106.141986

[25] Kingston PA, Sinha S, Appleby CE, David A, Verakis T, et al (2003) Adenovirus-mediated gene transfer of transforming growth factor-beta3, but not transforming growth factor-beta1, inhibits constrictive remodeling and reduces luminal loss after coronary angioplasty. Circulation 108: 2819–2825. 14638551 10.1161/01.CIR.0000097068.49080.A0

[26] Nugent HM, Rogers C, Edelman ER (1999) Endothelial implants inhibit intimal hyperplasia after porcine angioplasty. Circ Res 84: 384–391. 10066672 10.1161/01.res.84.4.384

[27] Tian W, Kuhlmann MT, Pelisek J, Scobioala S, Quang TH, et al (2006) Paclitaxel delivered to adventitia attenuates neointima formation without compromising re-endothelialization after angioplasty in a porcine restenosis model. Journal of endovascular therapy: an official journal of the International Society of Endovascular Specialists 13: 616–629. 17042659 10.1583/05-1802MR.1

[28] Brown MA, Zhang L, Levering VW, Wu JH, Satterwhite LL, et al (2010) Human umbilical cord blood-derived endothelial cells reendothelialize vein grafts and prevent thrombosis. Arterioscler Thromb Vasc Biol 30: 2150–2155. 20798381 10.1161/ATVBAHA.110.207076 PMC2959120

[29] Antczak C, Shum D, Escobar S, Bassit B, Kim E, et al (2007) High-throughput identification of inhibitors of human mitochondrial peptide deformylase. Journal of biomolecular screening 12: 521–535. 17435169 10.1177/1087057107300463 PMC2234356

[30] Sachsenmeier KF, Hay C, Brand E, Clarke L, Rosenthal K, et al (2012) Development of a novel ectonucleotidase assay suitable for high-throughput screening. Journal of biomolecular screening 17: 993–998. 22522649 10.1177/1087057112443987

[31] Wessely R, Schomig A, Kastrati A (2006) Sirolimus and Paclitaxel on polymer-based drug-eluting stents: similar but different. J Am Coll Cardiol 47: 708–714. 16487832 10.1016/j.jacc.2005.09.047

[32] Hofma SH, van der Giessen WJ, van Dalen BM, Lemos PA, McFadden EP, et al (2006) Indication of long-term endothelial dysfunction after sirolimus-eluting stent implantation. European heart journal 27: 166–170. 16249221 10.1093/eurheartj/ehi571

[33] Torella D, Gasparri C, Ellison GM, Curcio A, Leone A, et al (2009) Differential regulation of vascular smooth muscle and endothelial cell proliferation in vitro and in vivo by cAMP/PKA-activated p85alphaPI3K. Am J Physiol Heart Circ Physiol 297: H2015–2025. 19783773 10.1152/ajpheart.00738.2009

[34] Song P, Wang S, He C, Liang B, Viollet B, et al (2011) AMPKalpha2 deletion exacerbates neointima formation by upregulating Skp2 in vascular smooth muscle cells. Circ Res 109: 1230–1239. 21980125 10.1161/CIRCRESAHA.111.250423 PMC3235405

[35] Li FY, Lam KS, Tse HF, Chen C, Wang Y, et al (2012) Endothelium-selective activation of AMP-activated protein kinase prevents diabetes mellitus-induced impairment in vascular function and reendothelialization via induction of heme oxygenase-1 in mice. Circulation 126: 1267–1277. 22851545 10.1161/CIRCULATIONAHA.112.108159

[36] Ewald JA, Peters N, Desotelle JA, Hoffmann FM, Jarrard DF (2009) A high-throughput method to identify novel senescence-inducing compounds. Journal of biomolecular screening 14: 853–858. 19641224 10.1177/1087057109340314 PMC2913693

[37] Tomasini-Johansson BR, Johnson IA, Hoffmann FM, Mosher DF (2012) Quantitative microtiter fibronectin fibrillogenesis assay: use in high throughput screening for identification of inhibitor compounds. Matrix biology: journal of the International Society for Matrix Biology. 10.1016/j.matbio.2012.07.003 PMC3508085 22986508

[38] Mukadam S, Tay S, Tran D, Wang L, Delarosa EM, et al (2012) Evaluation of time-dependent cytochrome p450 inhibition in a high-throughput, automated assay: introducing a novel area under the curve shift approach. Drug metabolism letters 6: 43–53. 22372554 10.2174/187231212800229309

[39] Volkova M, Russell R 3rd (2011) Anthracycline cardiotoxicity: prevalence, pathogenesis and treatment. Current cardiology reviews 7: 214–220. 22758622 10.2174/157340311799960645 PMC3322439

[40] Gertz ZM, Wilensky RL (2011) Local drug delivery for treatment of coronary and peripheral artery disease. Cardiovascular therapeutics 29: e54–66. 20553281 10.1111/j.1755-5922.2010.00187.x

[41] Werk M, Langner S, Reinkensmeier B, Boettcher HF, Tepe G, et al (2008) Inhibition of restenosis in femoropopliteal arteries: paclitaxel-coated versus uncoated balloon: femoral paclitaxel randomized pilot trial. Circulation 118: 1358–1365. 18779447 10.1161/CIRCULATIONAHA.107.735985

[42] Cremers B, Schmitmeier S, Clever YP, Gershony G, Speck U, et al.. (2013) Inhibition of neo-intimal hyperplasia in porcine coronary arteries utilizing a novel paclitaxel-coated scoring balloon catheter. Catheterization and cardiovascular interventions: official journal of the Society for Cardiac Angiography & Interventions. 10.1002/ccd.25296 24259380

