# Correction: High-Throughput Screening Identifies Idarubicin as a Preferential Inhibitor of Smooth Muscle versus Endothelial Cell Proliferation

**DOI:** 10.1371/journal.pone.0110098

**Published:** 2014-10-01

**Authors:** The PLOS ONE Staff

The following information is missing from the Funding section: This work was supported by University of Wisconsin ICTR-Clinical and Type 1 Translational Research Pilot Program (UL1TR000427 to K.C.K.). This funder had no role in study design, data collection and analysis, decision to publish, or preparation of the manuscript.
