# Supplementary figures and images for: High-Throughput Screening Identifies Idarubicin as a Preferential Inhibitor of Smooth Muscle versus Endothelial Cell Proliferation
Source: PLoS One. 2014 Feb 24;9(2):e89349. doi: 10.1371/journal.pone.0089349 (PMC3933427; doi:10.1371/journal.pone.0089349)

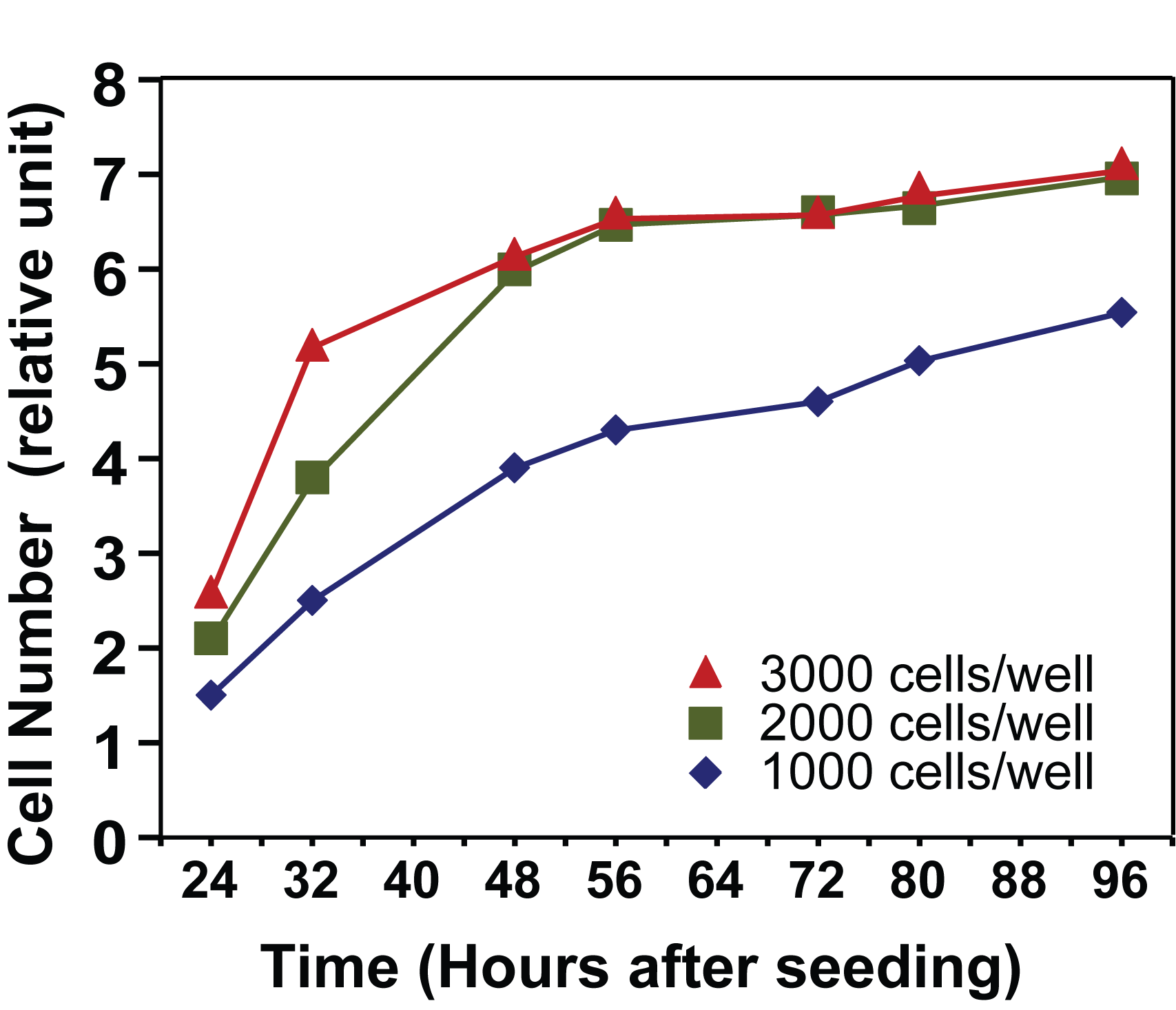

Supplement: Figure S1 — Time courses of the growth of HuAoSMCs seeded at different densities. SMCs were seeded at 1000 (blue), 2000 (green), or 3000 (red) cells/well on a 96-well plate, and cultured in SmGM-2 supplemented with 5% serum. Alamar Blue dye was added at different time points (to separate wells) and after a 24 h continued incubation fluorescence was read. A background reading from cell-free wells was subtracted. (TIF) [file pone.0089349.s001.tif]

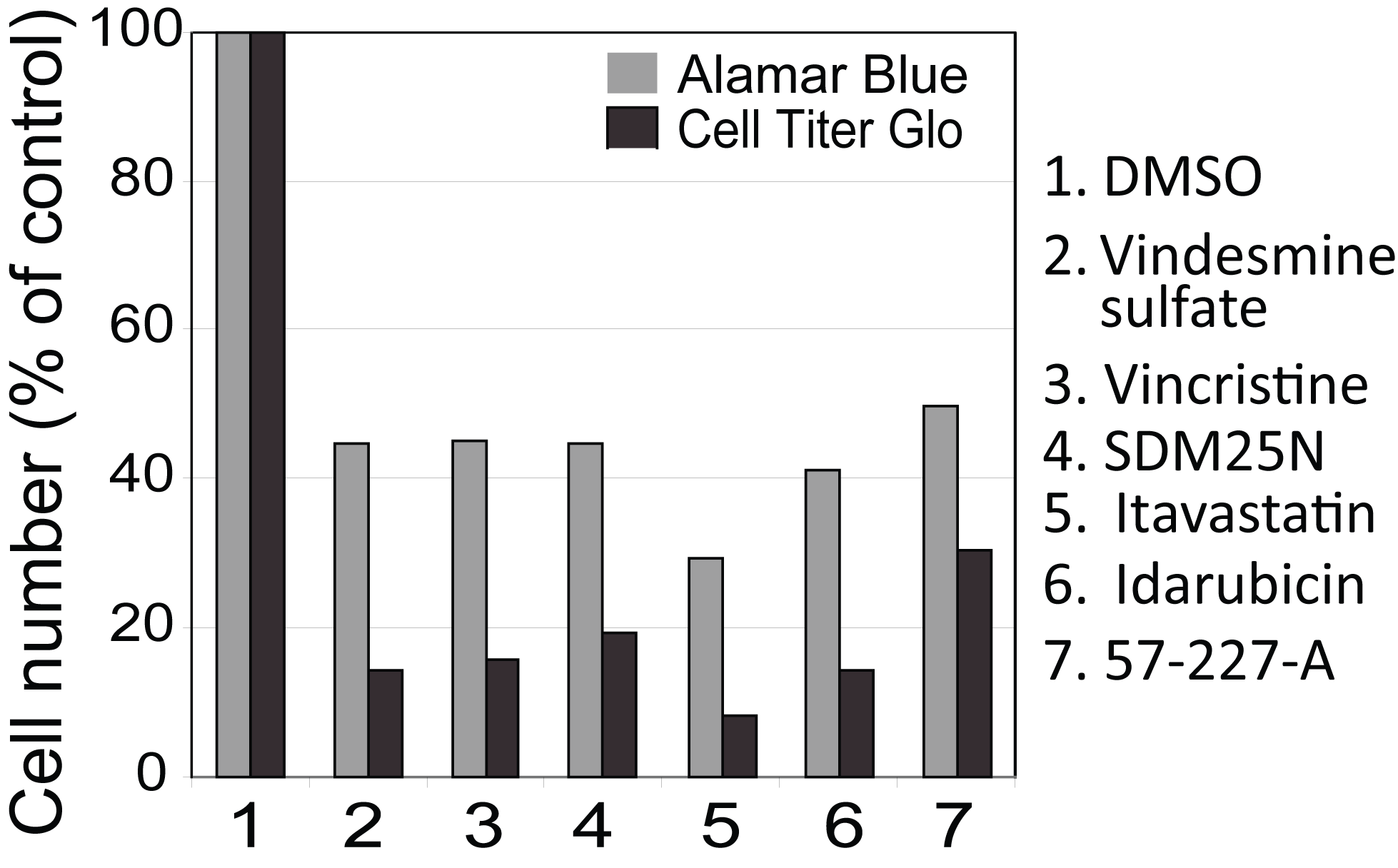

Supplement: Figure S2 — Re-test of the initial hits from the HTS using Cell Titer Glo assay. Following the HTS assay of HuAoSMC proliferation, Alamar Blue dye was removed and the wells were gently washed by the automated system. The plates were then subjected to Cell Titer Glo assay, and percent inhibition of SMC proliferation by some of the initial 11 hits was compared between these two different assay methods. (TIF) [file pone.0089349.s002.tif]
